# Clinical characteristics, treatment course and outcome of adults treated for avoidant/restrictive food intake disorder (ARFID) at a tertiary care eating disorders program

**DOI:** 10.1186/s40337-024-00973-6

**Published:** 2024-01-23

**Authors:** Danielle E. MacDonald, Rachel Liebman, Kathryn Trottier

**Affiliations:** 1 Centre for Mental Health, University Health Network, 7-Eaton South, 200 Elizabeth Street, Toronto, ON M3J 2Z3 Canada; 2 Department of Psychiatry, University of Toronto, 250 College Street, 8th Floor, Toronto, ON M5T 1R8 Canada; 3 Department of Psychology, Toronto Metropolitan University, 350 Victoria Street, Toronto, ON M5B 2K3 Canada

**Keywords:** ARFID, Avoidant/restrictive food intake disorder, Cognitive behaviour therapy, CBT-AR, Inpatient, Intensive outpatient, Retrospective design

## Abstract

**Background:**

Avoidant/restrictive food intake disorder (ARFID) is now recognized as a feeding/eating disorder that affects individuals across the lifespan, but research on ARFID in general and particularly in adults remains limited. The purpose of this study was to describe the demographic and clinical characteristics of adults with ARFID seeking treatment at a tertiary care eating disorders program, and to describe the course and outcomes of treatment at three levels of care—inpatient, intensive outpatient, and outpatient individual therapy.

**Method:**

This retrospective chart review study examined the charts of 42 patients who received treatment for ARFID between April 2020 and March 2023. Following diagnostic assessment, patients were referred to either inpatient treatment, intensive outpatient treatment, or outpatient individual therapy. All three levels of care involved individual cognitive behaviour therapy. Inpatients typically transitioned to one of the outpatient treatments as part of a continuous care plan. We examined demographic and clinical characteristics, treatment length and completion, and changes in key indicators during treatment.

**Results:**

Patients were diverse with respect to demographics (e.g., 62% cisgender women; 21% cisgender men; 17% transgender, non-binary, or other gender) and comorbid concerns (e.g., 43% had neurodevelopmental disorders; > 50% had mood and anxiety disorders; 40% had posttraumatic stress disorder [PTSD]; 35% had medical conditions impacting eating/digestion). Most patients presented with more than one ARFID maintaining mechanism (i.e., lack of appetite/interest, sensory sensitivities, and/or fear of aversive consequences of eating). Treatment completion rates and outcomes were good. On average, patients showed significant improvement in impairment related to their eating disorder, and those who were underweight significantly improved on BMI and were not underweight at end of treatment.

**Discussion:**

These findings add to the literature by indicating that ARFID patients are commonly male or have diverse gender identities, and have high rates of neurodevelopmental, mood, anxiety, and gastrointestinal disorders. We also found high rates of PTSD. The findings show promise for treatment outcomes across the continuum of care. Next steps in ARFID treatment and research include incorporating ARFID-specific assessments into routine care, and ongoing research investigating the efficacy and effectiveness of treatments such as CBT-AR.

## Abstract (plain language summary)

Avoidant/restrictive food intake disorder (ARFID) is now recognized as a feeding/eating disorder that affects individuals across the lifespan, but research on ARFID in general and particularly in adults remains limited. The purpose of this study was to describe the demographic and clinical characteristics of adults with ARFID seeking treatment at a tertiary care eating disorders program, and to describe the course and outcomes of treatment at three levels of care—inpatient, intensive outpatient, and outpatient individual therapy.

## Background

Avoidant/restrictive food intake disorder (ARFID) is characterized by inadequate food intake that is associated with significant physical and/or psychosocial consequences and is *not* due to weight or shape concerns. Rather, in ARFID, food avoidance and/or restriction is related to lack of appetite or interest in eating, sensory aversions (i.e., related to the taste, texture, odor, or appearance of food), and/or fear of aversive consequences of eating (e.g., choking, vomiting) [1]. Individuals with ARFID are often significantly underweight, may have substantial nutritional deficiencies, and may be dependent on oral supplements or tube feeding [1]. ARFID was previously classified as a feeding/eating disorder limited to infancy and early childhood [2]. More recently, it has become clear that ARFID can affect individuals across the age spectrum, from early childhood to adulthood. As a result of this increased recognition in older youth and adults, the diagnostic criteria for ARFID were revised in the *Diagnostic and Statistical Manual of Mental Disorders, fifth edition* (DSM-5) to reflect that the illness can occur across the lifespan [1]. Epidemiological research on ARFID is in its infancy, and there has been significant methodological and sampling variability between the studies to date, meaning the true prevalence of ARFID in the general population and in clinical settings is not yet known [3]. In the ten years since the DSM-5 formally recognized ARFID as affecting individuals of all ages, clinical recognition and research on assessment, classification, and treatment has grown, yet remains limited, particularly in adults.

A systemic scoping review that included patients across the lifespan concluded that ARFID is a valid clinical syndrome distinct from other eating disorders, but that the extant research is sparse in all areas of investigation [4]. With respect to their clinical presentation, individuals with ARFID often present with more than one ARFID maintaining mechanism (i.e., lack of appetite/interest in eating, sensory sensitivities, and fear of aversive consequences of eating, which reflect the three primary sets of concerns that typically maintain ARFID psychopathology) [3, 4]. Earlier research indicated that ARFID may be more likely to occur in boys and men than in girls and women [4], but more recent research suggests that the sex distribution of ARFID may be relatively equal [3, 5]. Commonly comorbid neurodevelopmental and psychiatric disorders include attention deficit hyperactivity disorder (ADHD), autism spectrum disorder (ASD), and anxiety disorders [3, 4]. Mood disorders are also frequently experienced by individuals with ARFID [3, 6], although the available research suggests that they may be less commonly comorbid with ARFID than with other eating disorders [4]. Obsessive–compulsive disorder and internet gaming disorder have also been found to co-occur with ARFID, though the rates of co-occurrence are not yet clear [3, 4]. It is not clear how often individuals with ARFID experience comorbid posttraumatic stress disorder (PTSD) [3, 4], though one recent study reported that 20% of the sample had a trauma- or stressor-related disorder [6]. Gastrointestinal disorders and complaints are the most frequently reported co-occurring medical concern among patients with ARFID [6]. Importantly, most of the research on ARFID has been conducted on children and adolescents, and the extant research focused on adults is extremely limited [3, 4, 7, 8].

The literature on treatment of ARFID in adults has largely consisted of single patient case studies (e.g., [9–12]) and case series of youth (including children, adolescents, and young adults) (e.g., [13–16]). The treatments utilized with adults and described in these papers are diverse, and include individual cognitive behavior therapy (CBT) delivered in inpatient [11, 17] and outpatient [10, 12] contexts, CBT-based day treatment [14], “temperament-based treatment” for young adults [18], other unspecified psychotherapy [9], psychotropic medications [10, 13, 15], and nutritional rehabilitation [16, 17].

Most recently, a CBT protocol specifically for ARFID (CBT-AR) [19] has been tested in both youth and adults and has shown promise in improving symptoms of ARFID. CBT-AR is a 20- to 30-session structured psychotherapy for children, adolescents, and adults that targets core ARFID symptoms and maintaining mechanisms [19]. An initial uncontrolled trial of CBT-AR in 15 adults (and the first known prospective treatment study of ARFID in adults) found strong treatment acceptability, good completion rates, and significant improvements on clinical indicators from pre- to post-treatment [20]. Nearly half of patients were considered fully remitted by end-of-treatment, and irrespective of remission status, clinicians rated 80% of the sample as much improved in their symptoms from pre- to post-treatment [20]. Another CBT protocol specifically for individuals with ARFID and functional gastrointestinal disorders (i.e., gastrointestinal problems with no underlying structural abnormality) was recently developed and examined in 14 adults in routine clinical practice, and results showed improvements in ARFID-related fears [21].

Although the literature on treatment of ARFID in adults is emerging, there is still much to be learned about the types of patients presenting for treatment and about the course and outcomes of treatment, including both CBT-AR and other approaches.

## The current study

The purpose of this study was to contribute to the emerging literature by describing the demographic and clinical profiles and treatment outcomes of treatment-seeking adults with ARFID. We sought to examine the demographic, eating disorder-related, and comorbid concerns experienced by this group. Given that PTSD is common in individuals with other eating disorders [22] but little is known about whether this extends to ARFID, we sought to examine the co-occurrence of ARFID and PTSD. Finally, the study aimed to contribute to the burgeoning treatment literature by describing the outcomes of ARFID-specific treatment at three levels of care—inpatient treatment, intensive outpatient treatment, and individual therapy.

### Research questions

1. What are the clinical and demographic characteristics of adult ARFID patients treated at a tertiary care eating disorder program in the Canadian public healthcare system? Specifically:
  - Demographic characteristics: Age; gender; race and ethnicity; marital status; occupational status; financial independence versus dependence; and education level.
  - Clinical characteristics: ARFID maintaining mechanism(s); Body mass index (BMI); co-occurring psychiatric and medical concerns; proportion of the sample with current PTSD symptoms; “classic” eating disorder psychopathology (i.e., eating and weight/shape concerns typical of those with anorexia nervosa, bulimia nervosa, and other similar eating disorders); and degree of impairment due to ARFID.
2. What are the characteristics of treatment course and end-of-treatment outcomes for adults with ARFID treated at different levels of care? Specifically:
  - Treatment course: length; rate of completion; and rate of transition from inpatient to outpatient treatment (when relevant).
  - End-of-treatment outcomes:
    i. Change in body mass index (BMI; if significantly underweight [BMI < 17.5] at start of treatment).
    ii. Change in clinical impairment associated with ARFID.

## Methods

### Design and participants

This study used a retrospective chart review to identify eligible patients during the study period. Eligible participants: (1) had a DSM-5 diagnosis of ARFID; and (2) started treatment at the University Health Network’s Eating Disorder Program between April 2020 and March 2023 (which aligns with when our program began offering services for patients with ARFID). Clinical services and data collection occurred as part of routine clinical care; the University Health Network Research Ethics Board approved access to and retrospective use of these data for this study.

### Treatments

Following referral to the program, a psychologist or psychiatrist saw patients for assessment of their eating disorder-related concerns. The assessment involved a semi-structured clinical interview based on DSM-5 criteria developed for the program, and which resulted in a DSM-5 eating disorder diagnosis, as applicable. Our program offered three potential levels of care for patients with ARFID during the study period: inpatient treatment; intensive outpatient treatment (IOP); and outpatient individual therapy. Level of care decisions were based on clinical indication. Inpatient treatment was recommended when individuals had a BMI < 16; there was a high risk for refeeding syndrome; there were other medical or psychiatric concerns warranting hospitalization; the severity of the patient’s eating disturbance required a high level of supervision and support to facilitate regular eating and prevent eating disorder behaviours; or other reasons as clinically indicated. IOP was typically recommended for individuals with a BMI < 18.5 and/or for those who had not responded to outpatient individual therapy or were otherwise thought to require more frequent and structured support to achieve regular eating and other treatment goals. Otherwise, patients were referred to outpatient individual therapy.

Inpatient treatment for patients with ARFID involved a hospital admission to a specialized inpatient eating disorders service. Standard benchmark admission lengths were 6 weeks for patients who were severely underweight (BMI < 16) at admission and 3 weeks for others. Actual admission lengths varied based on clinical indication. A multidisciplinary care team (i.e., psychologists, psychiatrists, nurse practitioners and nurses, social workers, registered dietitians, occupational therapists, and registered psychotherapists) delivered the inpatient treatment. Treatment included: 5–6 supervised meals and snacks per day; individual CBT sessions several times per week; group psychotherapy several times per week; individual sessions with occupational therapy, social work, and nutrition; care partner meetings when indicated; and medical and psychiatric care. Treatment targets included: establishing a regular pattern of eating including adequate, varied nutrition; weight restoration (when indicated); and making initial changes to ARFID-related concerns. Although inpatient treatment targeted these clinical concerns for all patients as relevant, specific nutritional, weight-related, and behavioural goals were individualized. Inpatient treatment was intended to be followed by transition to outpatient treatment, where patients learned to generalize the changes made in hospital to their home environment, continued to improve eating and weight (when relevant), and addressed ARFID-related maintaining mechanisms through CBT-AR.

IOP and individual therapy patients received CBT-AR [19], which was delivered in accordance with the manual. Sessions were planned to be twice per week for the first 16 sessions and once per week for the final 4 sessions.^1^ Patients in the individual therapy program received CBT-AR on its own, with no other programming. Patients in the IOP program received CBT-AR plus 4–8 weeks of concurrent group-based programming. The group programming consisted of one clinician-supported meal and one psychotherapy group per day, from Monday to Friday. Psychotherapy groups included two CBT skills groups, two DBT skills groups (adapted from Linehan [23]), and one eating skills group per week. IOP patients also received sessions with a registered dietitian and/or occupational therapist if clinically indicated. The outpatient treatments were delivered via videoconference due to COVID-19 pandemic restrictions.

### Measures

#### Eating Disorder Examination 16.0 (EDE)

The EDE is a clinician-administered structured clinical interview that assesses cognitive and behavioural eating disorder psychopathology over the prior three months [24]. It yields four subscales as well as a Global Score that reflects overall eating disorder psychopathology related to anorexia nervosa, bulimia nervosa, binge-eating disorder, and related eating disorders. Psychometric properties are good in patients with these types of eating disorders [25]. To our knowledge, there are no published psychometrics on the EDE in adults with ARFID. Cronbach’s alpha for the Global score at baseline in the present sample was α = 0.78.

#### Clinical Impairment Assessment Questionnaire 3.0 (CIA)

The CIA is a 16-item self-report questionnaire assessing functional impairment due to an eating disorder [26]. Items are scored on a 4-point Likert scale ranging from 0–3, and it produces a global score reflecting global impairment. The CIA global score has good psychometric properties in non-ARFID eating disorder samples [27]. To our knowledge, there are no published psychometrics on the CIA in adults with ARFID. The CIA has good accuracy in differentiating eating disorders from control cases, with a cut-off score > 16 to classify significant clinical impairment due to an eating disorder [28]. Cronbach’s alpha at baseline in the present sample was α = 0.96.

#### PTSD Checklist-5 (PCL-5)

The PCL-5 is a 20-item self-report questionnaire assessing symptoms of PTSD consistent with DSM-5 criteria [29]. It assesses the presence and nature of a past traumatic event, as well as the severity of current PTSD symptoms related to this event, which are rated on a 5-point Likert scale ranging from 0–4. It has good psychometric properties [30]. Cronbach’s alpha at baseline in the present sample was α = 0.95. We examined PCL scores in two ways:

1. The PCL-5 was used to classify whether individuals screened positive for PTSD, based on both of the following: (a) they listed an event consistent with a DSM-5 criterion A traumatic event, and (b) met the DSM-5 diagnostic symptom count criteria [1, 29].
2. Continuous PCL-5 scores were used to examine PTSD symptom severity in individuals who had reported an event consistent with a DSM-5 criterion A traumatic event [29].

### Procedure

The study assessment measures (i.e., EDE, CIA, and PCL-5) were administered as part of a clinical intake assessment at start of treatment, and the EDE and CIA were administered again at end of each treatment service. Weight and height were measured at the start of treatment and weight was measured throughout treatment. Diagnosis, ARFID maintaining mechanisms, and demographic and clinical information gathered at initial diagnostic assessment, intake assessment, throughout treatment, and at end-of-treatment were examined retrospectively for the purpose of the study. Charts were also reviewed for documentation of comorbid mental health or neurodevelopmental diagnoses either diagnosed as part of the assessment in our program or noted in the assessment report as a prior diagnosis (i.e., previously documented in the patient’s clinical record or reported by the patient).

### Statistical analyses

Statistical analyses were conducted in SPSS version 24.0. Descriptive statistics were used to examine and describe the characteristics of the sample and the treatments. A one-sample *t* test was used to compare EDE Global scores to the community mean. Paired samples *t* tests were used to examine changes in BMI, weight, and CIA scores from pre- to post-treatment. A value of *p* < 0.05 was used to indicate statistical significance.

## Results

### Characteristics of the sample

A total of 42 unique patients with ARFID participated in treatment in our program during the study period. Individuals who transitioned from inpatient to outpatient treatment were considered as having one single course of treatment. There were 43 unique courses of treatment: One individual had two courses of treatment, and the remainder (97.6%) were unique. For the individual who attended treatment twice, the most recent course of treatment was used for data analyses. See Table 1 for demographic characteristics of the sample.

**Table 1:** Demographic characteristics (N = 42)

Variable	M (SD) or %	n
Age	26.0 (6.9)	42
Gender		
Cisgender woman	61.9%	26
Cisgender man	21.4%	9
Transgender, non-binary, or other gender identity	16.7%	7
Race and ethnicity (n = *41)*		
White	65.9%	27
Asian	7.3%	3
Indigenous	4.9%	2
Black, middle eastern, or other	17.1%	7
Biracial/multi-racial	4.9%	2
Marital status (n = *41)*		
Single	90.2%	37
Married or common-law partnership	7.3%	3
Separated or divorced	2.4%	1
Currently employed (*n* = 41)	31.7%	13
Currently a student (*n* = 41)	24.4%	10
Financial support (n = *39)*		
Self-supporting	23.1%	9
Partially self-supporting	33.3%	13
Completely dependent (on partner, family, or government)	43.6%	17
Educational attainment (n = *41)*		
Below high school	22.0%	9
High school	41.5%	17
College diploma	14.6%	6
Undergraduate degree	14.6%	6
Professional or graduate degree	7.3%	3

We combined some categories with small *n*s strictly to ensure de-identification. There is no inference by the authors that the combined identity categories represent the same group

With respect to demographic characteristics, the gender breakdown of the study sample was diverse, with 61.9% identifying as cisgender women, 21.4% identifying as cisgender men, and 16.7% identifying as transgender, gender non-binary, or another diverse gender identity. The sample ranged in age from 17 to 48 years old, with an average age of 26.0 years (*SD* = 6.9), and most were single (90.2%). The majority were not employed (68.3%) or enrolled in school (75.6%), and most (76.9%) received some government or other financial support (with more than 40% of the sample fully financially dependent). The majority of the sample had a maximum educational attainment of high school (41.5%) or less than high school (22.0%).

The patients exhibited diverse presentations of the typical ARFID maintaining mechanisms. Of the total sample, 47.6% had the lack of appetite/interest presentation, 57.1% had the sensory sensitivities presentation, and 69.0% had the fear of aversive consequences. The majority (61.9%) exhibited two or more of the maintaining mechanisms. See Table 2 for further details.

**Table 2:** ARFID maintaining mechanisms in the study sample (N = 42)

ARFID maintaining mechanism(s)	%	*n*
Lack of appetite/interest *only*	4.8%	2
Sensory sensitivities *only*	4.8%	2
Fear of aversive consequences *only*	28.6%	12
Lack of appetite/interest and sensory sensitivities	21.4%	9
Lack of appetite/interest and fear of aversive consequences	9.5%	4
Sensory sensitivities and fear of aversive consequences	19.0%	8
All 3 (lack of appetite/interest, sensory sensitivities, and fear)	11.9%	5

Patients with the fear of aversive consequences mechanism reported a diverse array of feared outcomes related to eating, including: vomiting, nausea, gagging; foodborne illness; gastrointestinal pain or distress; bloating or other aversive sensory concerns due to eating; choking or problems swallowing; allergic reaction

Our sample had a diverse array of psychiatric comorbidities, including: Neurodevelopmental disorders (42.9%); mood disorders (52.4%); anxiety disorders (61.9%); obsessive compulsive disorder (16.7%); trauma- and stressor-related disorders (21.4%); somatic symptom disorder (7.1%); substance use disorder (4.8%); and borderline personality disorder (9.5%). A subset of patients (*n* = 15; 35.7%) were also documented as having one or more comorbid medical condition that may affect food, eating, digestion, or their gastrointestinal system (e.g., Crohn’s disease, Celiac disease, GERD). See Table 3 for further details.

**Table 3:** Co-occurring psychiatric and medical concerns (N = 42)

Comorbid diagnosis	%	*n*
*Psychiatric*	90.5	38
Neurodevelopmental disorders	42.9	18
Autism spectrum disorder	16.7	7
Attention deficit hyperactivity disorder	26.2	11
Learning disability or intellectual disability	14.3	6
Mood disorders	52.4	22
Major depressive disorder (or other similar depressive disorder)	47.6	20
Bipolar disorder	4.8	2
Anxiety disorders	61.9	26
Generalized anxiety disorder	50.0	21
Social anxiety disorder	16.7	7
Other Anxiety Disorder^a^	16.7	7
Unspecified anxiety disorder	4.8	2
Obsessive compulsive disorder	16.7	7
Posttraumatic stress disorder or other trauma/stressor-related disorder	21.4	9
Substance use disorder	4.8	2
Borderline personality disorder	9.5	4
Somatic symptom disorder	7.1	3
*Medical*	35.7	15
Celiac disease	7.1	3
Crohn’s disease	7.1	3
GERD	7.1	3
Food allergies	7.1	3
Other gastrointestinal disorders	3.1	3
Other conditions affecting food or eating	9.5	4

Comorbid concerns were obtained from chart review based on either what was diagnosed following assessment in our program, or noted at assessment as a pre-existing diagnosis within the clinical record or self-reported by the patient. We combined some categories with small *n*s to ensure de-identification

^a^“Other anxiety disorder” included individuals diagnosed with any of the following: specific phobia, panic disorder, agoraphobia, or selective mutism

Given possible etiological differences for patients with either the lack of appetite/interest of sensory sensitivities mechanisms compared to the fear of aversive consequences mechanism of ARFID [19], we also descriptively examined the rates of neurodevelopmental and anxiety disorders in these two groups (as these concerns may be particularly relevant to understanding possible etiological differences). In the participants with exclusively the lack of appetite and/or sensory sensitivities presentation, 46.2% had neurodevelopmental disorders and 53.8% had anxiety disorders. In contrast, in participants with exclusively the fear of aversive consequences presentation, 25.0% had neurodevelopmental disorders whereas 50.0% had anxiety disorders.

### Treatments received

**Fig. 1:**
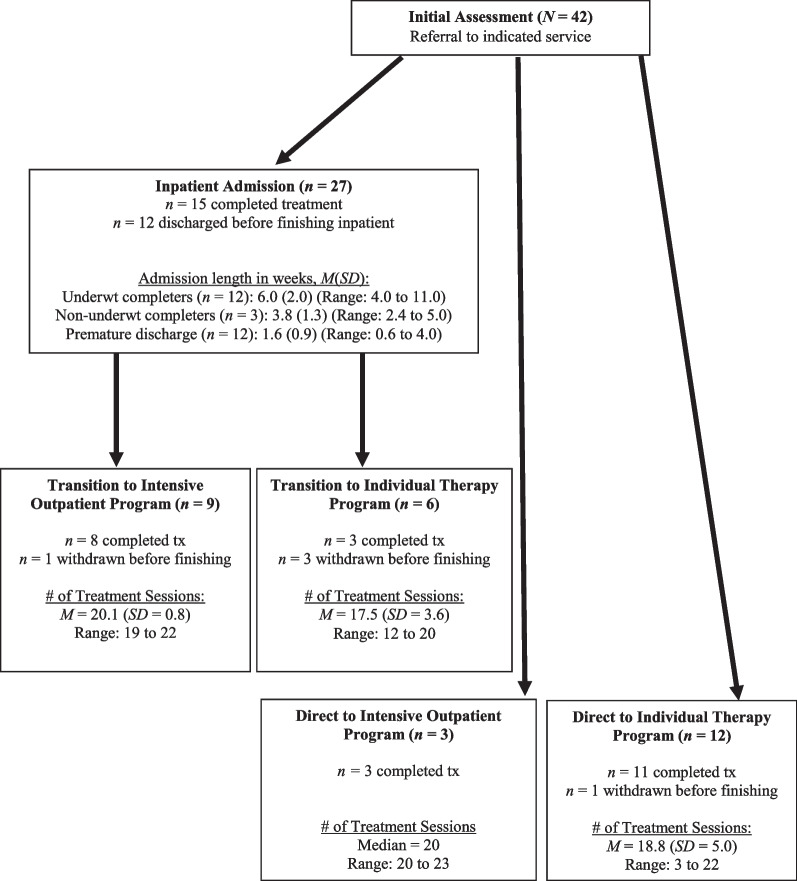
Treatment flow diagram including length of treatment, by treatment type

**Fig. 2:**
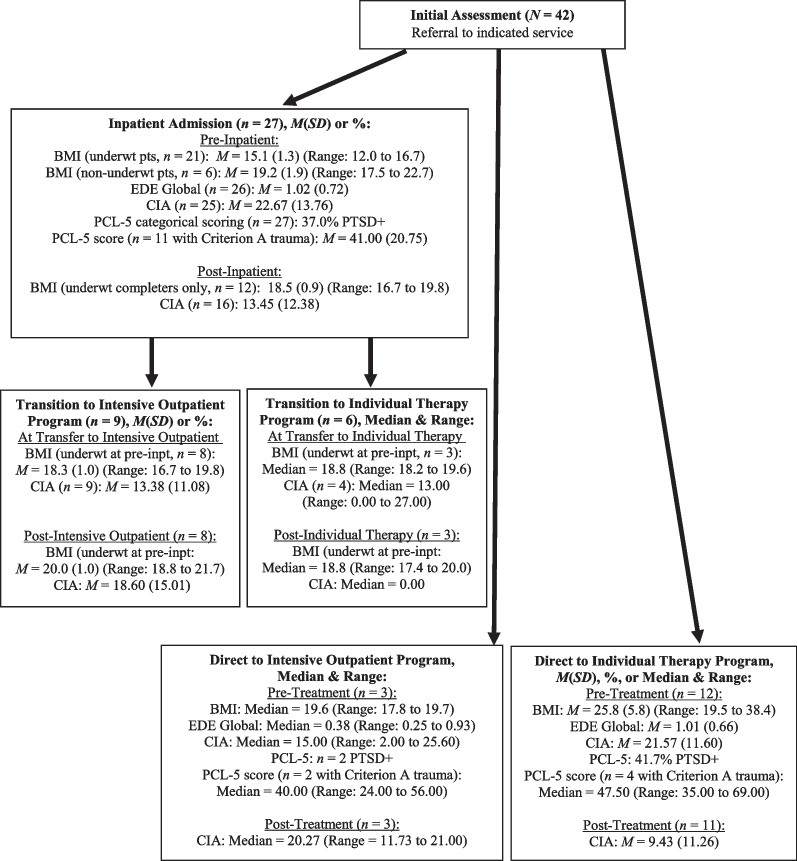
Pre-treatment and post-treatment clinical variables, by treatment type

See Fig. 1 for a treatment flow diagram that maps out the patients’ flow through treatment, including completion rates and length of treatment. See Fig. 2 for an additional treatment flow diagram that summarizes pre-treatment clinical variables and post-treatment clinical outcomes (i.e., BMI, EDE Global, CIA, and PCL-5 scores), by treatment type. Note that in Figs. 1 and 2, some of the outpatient treatment cells/results had small sample sizes (i.e., *n*s = 3 to 4). In these instances, medians and ranges are reported in Fig. 1 and 2 instead of means and standard deviations or percentages (as applicable). Means, standard deviations, and percentages for the combined outpatient groups (i.e., IOP and individual therapy, and reported separately for inpatient transfers to outpatient versus direct entry to outpatient) are presented in the main text as indicated.

#### Inpatient treatment

Of the 42 unique patients in the study, 27 (64.3%) received inpatient treatment. Dispositions at end of inpatient treatment for these 27 patients were: eight completed and transitioned to the IOP (29.6% of inpatients); five completed and transitioned to CBT-AR only (18.5% of inpatients); two ended inpatient treatment prematurely and transitioned to outpatient services (i.e., one to CBT-AR only and one to IOP; 2.7% of inpatients); and 12 (44.4%) did not transition to outpatient services. Of the 12 individuals who did not transition to outpatient treatment: two completed inpatient treatment and no further ARFID treatment was recommended; and 10 (37.0% of the total inpatients) were discharged prematurely. Of the 12 patients who ended treatment early (10 patient-initiated and two program-initiated discharges), nine patients had difficulty tolerating the inpatient environment or engaging with treatment and three believed the treatment was not a good fit to their needs.

 Of the nine patients who transitioned from inpatient to IOP, eight (88.9%) completed IOP and one (11.1%) ended treatment early. For the six patients who transitioned from inpatient to individual therapy only, three (50.0%) completed CBT-AR, and three (50.0%) ended treatment early. Reasons for withdrawal from the two outpatient services included problems with attendance, competing health concerns that interfered with treatment, and patient belief that they had achieved all their treatment goals prior to the end of the treatment protocol.

#### Direct entry to outpatient treatment

Fifteen of the 42 unique patients in the study started their course of treatment as outpatients. Three patients (7.1% of the total sample) had a direct entry to IOP, all of whom (100%) completed treatment. Twelve patients (28.6% of the total sample) received individual therapy only. Of these, 11 (91.7%) completed treatment, and one (8.3%) was withdrawn due to low motivation and engagement.

### Weight and BMI

#### Inpatient treatment

The majority of individuals who were admitted as inpatients (*n* = 21; 77.8%) were significantly underweight (BMI < 17.5) at admission, with an admission BMI range of 12.0 to 16.7. A paired sample *t* test showed that for underweight patients who completed inpatient treatment (*n* = 12), BMI increased significantly from pre- to post-admission, *t*(11) = -8.91, *p* < 0.001, with a mean increase of 3.6 (*SD* = 1.4) BMI points. Underweight patients who completed the recommended course of treatment gained an average of 10.4 (*SD* = 4.6) kg during inpatient treatment (Length of stay: *M* = 6.0 weeks [*SD* = 2.0], Range = 4.0 to 11.0), *t*(11) = -7.78, *p* < 0.001.

For patients who were significantly underweight at admission and who transitioned from inpatient treatment to one of the two outpatient treatments (*n* = 11), the average BMI at the end of outpatient treatment was no longer in the underweight range. BMI at transition to outpatient treatment was *M* = 18.5 (*SD* = 0.9; Range = 16.7 to 19.8) and BMI at end of outpatient treatment was *M* = 19.7 (*SD* = 1.0; Range = 17.4 to 21.7). A paired sample *t* test showed that these patients increased an additional 1.21 BMI points during outpatient treatment, *t* (10) = -3.28, *p* = 0.008.

#### Direct entry to outpatient treatment

None of the patients who started treatment as an outpatient were significantly underweight (BMI < 17.5) at baseline. Therefore, BMI change over time was not examined.

### Other clinical characteristics

#### “Classic” eating disorder psychopathology

Pre-treatment EDE Global scores were low at baseline for both inpatients (*M* = 1.02, *SD* = 0.72, *n* = 26) and outpatients (*M* = 0.91, *SD* = 0.63*, n* = 15). Both group means were less than one standard deviation from the published mean in a community norm sample of women without eating disorders (i.e., *M* = 0.932*, SD* = 0.805, *N* = 243) [17]. Because baseline EDE global scores were not elevated, post-treatment EDE scores were not examined.

#### Impairment due to the eating disorder

Pre-treatment CIA global scores exceeded the published cut-off score of 16.0 for inpatients (*M* = 22.67, *SD* = 13.76) and outpatients (*M* = 20.10, *SD* = 11.61)*,* indicating significant clinical impairment due to the eating disorder [21]. Overall change in CIA scores during treatment (regardless of each patient’s specific treatment trajectory through one or more of the treatments) was examined using a paired samples *t* test for all patients who had baseline and post-treatment CIA data (*n* = 28). Over the course of treatment, patients made significant improvements in CIA scores, *t*(27) = 3.19, *p* = 0.004. The overall mean decreased from 20.38 (*SD* = 12.37) at baseline to 12.49 (*SD* = 12.86) at end-of-treatment, and 71.4% were below the threshold for clinically significant impairment.

#### Current trauma-related symptoms

Seventeen patients (40.5% of the sample) screened positive for PTSD at baseline using the PCL-5 (37.0% of the inpatient group and 46.7% of the outpatient group). Mean PCL-5 scores for those who reported a DSM-5 criterion A trauma were *M* = 41.00 (*SD* = 20.75, *n* = 11) for inpatients and *M* = 46.50 (*SD* = 15.76, *n* = 6) for outpatients. These scores reflect high levels of PTSD symptom severity.

## Discussion and conclusions

This study describes the characteristics of adults with ARFID presenting for treatment to a publicly funded tertiary care eating disorder program, as well as on the courses and outcomes of their treatment, an area that is lacking in the literature. With respect to the demographic characteristics of the patients, the group represented a diverse array of gender identities, and on average was relatively young. It is noteworthy that even adults presenting for ARFID treatment tend to be younger adults (in their mid-20 s on average), although this is similar to treatment-seeking individuals with other eating disorders in our setting (e.g., [31]). Additionally, the baseline CIA scores indicate clinically significant impairment due to the eating disorder for both the inpatient and outpatient groups, which highlights the substantial functional impairments experienced by our sample and is consistent with the diagnostic criteria for ARFID [1]. The low levels of employment and current enrollment in school, relatively low educational attainment, and significant rates of financial dependence *may* be further evidence of functional impairment in this group, although it is noted that this study is unable to assess the temporal relationship between these variables, and it is possible that they are simply correlated.

Additionally, comorbidity (psychiatric, neurodevelopmental, and medical) was common. In the present sample, the relatively high prevalence of neurodevelopmental disorders that typically emerge in childhood, including ASD, ADHD, and learning disorders, was striking and is consistent with prior research on ARFID [3, 4]. Likewise, we found high rates of anxiety disorders, which is also consistent with the literature on ARFID [3, 4]. Some research has indicated that children and adolescents with ARFID may be less likely to have mood disorders than those with other eating disorders [4] and that depression scores may be lower for individuals with ARFID than those with anorexia nervosa [32]. Other research has indicted that individuals with ARFID commonly experience co-occurring mood disorders [3, 6]. In support of this, more than half of patients in our sample reported a prior diagnosis of mood disorder. Mood and anxiety disorders are also commonly comorbid among those with “classic” (i.e., non-ARFID) eating disorders [33], suggesting potential similarity between these disorders and ARFID. Additionally, nearly 40% screened positive for PTSD symptoms at baseline, which is similar to the rate observed in patients with non-ARFID eating disorders presenting for treatment [22]. It has been postulated that PTSD symptoms likely play a mechanistic role in the maintenance of non-ARFID eating disorder psychopathology, for example via use of eating disorder symptoms such as restriction, binge eating, and/or purging to avoid trauma-related reminders [34]. This may suggest a similar relationship in ARFID and warrants further investigation.

Patients presented with all three of the ARFID-related mechanisms, and interestingly, most patients had at least two of the ARFID maintaining mechanisms simultaneously. This is consistent with prior research showing that a significant proportion of patients with ARFID exhibit more than one maintaining mechanism [35, 36] and showing that the three mechanisms are significantly inter-correlated [37]. In our sample, when only one mechanism was present, it was most often fear of aversive consequences, whereas lack of appetite and sensory sensitivities often co-occurred. This may reflect a different etiological pathway between the fear of aversive consequences presentation (which may present as a phobic-type reaction to a specific stimulus following a negative index event, such as a choking incident), compared to the lack of appetite/interest and sensory sensitivities mechanisms, which some experts have suggested may have biological contributors [19]. Interestingly, for the patients who exhibited the lack of appetite and/or sensory sensitivities mechanism (but not the fear of aversive consequences mechanism), 46% reported a prior diagnosis of neurodevelopmental disorder. In contrast, for those who exhibited *only* the fear of aversive consequences presentation, only 25% reported a neurodevelopmental disorder. Rates of anxiety disorders appeared more similar between these two ARFID groups. Although we did not compare these findings statistically due to small sample sizes, and although we cannot draw conclusions about these descriptive findings, our data echo prior research suggesting the possibility of unique etiological pathways between the different ARFID presentations (e.g., [38, 39]), and supports the need for further investigation of possible etiological and/or neurobiological differences between these groups. Understanding more about the etiology and maintenance of the different ARFID presentations may help further elucidate risk pathways and interventions targeting these concerns.

With respect to treatment, although the samples were small, treatment completion rates and outcomes were generally good. More than half of inpatients completed treatment, and premature withdrawal rates were similar to inpatient dropout rates for adults with anorexia nervosa [40]. More than 80% of all outpatients completed treatment. Additionally, underweight patients who received inpatient treatment were able to make significant gains with respect to weight restoration, and patients across all three levels of care exhibited improvements in the degree of impairment caused by the eating disorder.

### Strengths, limitations, and future directions

This study provides an important contribution to the burgeoning literature on ARFID, and in particular, provides a comprehensive description of the clinical characteristics and course of treatment for adults with ARFID presenting for treatment in a naturalistic, specialized eating disorder clinic at three different levels of care. These data contribute to our evolving understanding of the psychopathology of ARFID and of treatment approaches for ARFID in adults.

However, as a retrospective study, we were only able to describe and document what occurred in routine clinical practice. As a result, there is variability in assessment and treatment procedures, and we cannot draw conclusions about effectiveness of treatments from such data. Additionally, because we began assessing and treating ARFID in our clinic in an iterative manner in response to the clinical need, services evolved over time, and although ARFID diagnoses were based on DSM-5 criteria, our clinic does not utilize an ARFID-specific assessment measure. Nevertheless, we provided a detailed articulation of the clinical components of inpatient eating disorder treatment for adult patients with ARFID and observed good clinical outcomes, which may help to inform worthwhile components of treatment for individuals with the highest severity of ARFID. We are also able to contribute to the small but growing literature on CBT-AR and documented good outcomes from this treatment as delivered in routine practice. We also note that because we extracted the comorbid conditions from the chart review (which includes patient-reported prior diagnoses), these comorbidities must be interpreted cautiously, as there is likely variability in how these conditions were diagnosed. Nevertheless, these findings highlight the high rates of neurodevelopmental disorders among patients with ARFID, as well as some notable areas of overlap with classic eating disorders, which future research should investigate using more rigorous assessment methods. The current study provides naturalistic evidence that CBT-AR can be used successfully as both a standalone treatment and as part of an IOP treatment package. In the absence of randomized controlled trial data on CBT-AR (which is not yet available), our data add to the small but promising body of emerging evidence in support of this treatment.

Future directions to advance ARFID treatment research include use of ARFID-specific assessments in routine clinical care, such as the Pica, ARFID, and Rumination Disorder Interview (PARDI) [41], the Nine Item Avoidant/Restrictive Food Intake Disorder Screen (NIAS) [42], or the Food Neophobia Scale [43] in order to assess ARFID psychopathology comprehensively and in a standardized manner. Further evaluation of CBT-AR, including using a randomized controlled trial design, will be important to determining whether this is an efficacious treatment for ARFID. Finally, further research on understanding ARFID psychopathology—including the various ARFID mechanisms, the occurrence of ARFID-type mixed presentations, and the comorbidity profiles—may help further elucidate the psychopathology of ARFID.

## Data Availability

The dataset for this study cannot be made publicly available due to institutional ethical restrictions around patient confidentiality.

## Abbreviations

ADHD: Attention deficit hyperactivity disorder
ARFID: Avoidant/restrictive food intake disorder
ASD: Autism spectrum disorder
BMI: Body mass index
CBT: Cognitive behaviour therapy
CBT-AR: Cognitive behaviour therapy for avoidant/restrictive food intake disorder
CIA: Clinical Impairment Assessment Questionnaire
DSM-5: *Diagnostic and Statistical Manual of Mental Disorders, fifth edition*
EDE: Eating Disorder Examination
IOP: Intensive outpatient program
PCL-5: PTSD Checklist-5
PTSD: Posttraumatic stress disorder

## Footnotes

1 Typically, 20 sessions were offered per the standard CBT-AR protocol, though a small number of additional sessions were offered if clinically indicated. This is consistent with the protocol, which allows for variability in treatment length up to 30 sessions based on clinical need [19].

## References

[1] American Psychiatric Association (2013). Diagnostic and statistical manual of mental disorders.

[2] American Psychiatric Association (2000). Diagnostic and statistical manual of mental disorders.

[3] Archibald T, Bryant-Waugh R (2023). Current evidence for avoidant restrictive food intake disorder: implications for clinical practice and future directions. JCCP Adv.

[4] Bourne L, Bryant-Waugh R, Cook J, Mandy W (2020). Avoidant/restrictive food intake disorder: a systematic scoping review of the current literature. Psychiatry Res.

[5] Manwaring JL, Blalock DV, Rienecke RD, Le Grange D, Mehler PS (2023). A descriptive study of treatment-seeking adults with avoidant/restrictive food intake disorder at residential and inpatient levels of care. Eat Disord.

[6] Nitsch A, Watters A, Manwaring J, Bauschka M, Hebert M, Mehler PS (2023). Clinical features of adult patients with avoidant/restrictive food intake disorder presenting for medical stabilization: a descriptive study. Int J Eat Disord.

[7] Dalle Grave A, Sapuppo W (2020). Treatment of avoidant/restrictive food intake disorder: a systematic review. IJEDO.

[8] Willmott E, Dickinson R, Hall C, Sadikovic K, Wadhera E, Micali N, Trompeter N, Jewell T (2023). A scoping review of psychological interventions and outcomes for avoidant and restrictive food intake disorder (ARFID). Int J Eat Disord.

[9] Aloi M, Sinopoli F, Segura-Garcia C (2018). A case report of an adult male patient with avoidant/restrictive food intake disorder treated with CBT. Psychiatr Danub.

[10] Görmez A, Kilic A, Kirpinar I (2018). Avoidant/restrictive food intake disorder: an adult case responding to cognitive behavioral therapy. Clin Case Stud.

[11] King LA, Urbach JR, Stewart KE (2015). Illness anxiety and avoidant/restrictive food intake disorder: cognitive-behavioral conceptualization and treatment. Eat Behav.

[12] Mascarenhas Soffritti E, Calmeto Lomar Passos B, Rodrigues DG, de Freitas SR, PalazzoNazar B (2019). Adult avoidant/restrictive food intake disorder: a case report. J Bras Psiquiatr.

[13] Brewerton TD, D'Agostino M (2017). Adjunctive use of olanzapine in the treatment of avoidant restrictive food intake disorder in children and adolescents in an eating disorders program. J Child Adolesc Psychopharmacol.

[14] Dumont E, Jansen A, Kroes D, de Haan E, Mulkens S (2019). A new cognitive behavior therapy for adolescents with avoidant/restrictive food intake disorder in a day treatment setting: a clinical case series. Int J Eat Disord.

[15] Gray E, Chen T, Menzel J, Schwartz T, Kaye WH (2018). Mirtazapine and weight gain in avoidant and restrictive food intake disorder. J Am Acad Child Adolesc Psychiatry.

[16] Maginot TR, Kumar MM, Shiels J, Kaye W, Rhee KE (2017). Outcomes of an inpatient refeeding protocol in youth with anorexia nervosa: Rady Children's Hospital San Diego/University of California, San Diego. J Eat Disord.

[17] Makhzoumi SH, Schreyer CC, Hansen JL, Laddaran LA, Redgrave GW, Guarda AS (2019). Hospital course of underweight youth with ARFID treated with a meal-based behavioral protocol in an inpatient-partial hospitalization program for eating disorders. Int J Eat Disorder.

[18] Knatz Peck S, Towne T, Wierenga CE, Hill L, Eisler I, Brown T, Han E, Miller M, Perry T, Kaye W (2021). Temperament-based treatment for young adults with eating disorders: acceptability and initial efficacy of an intensive, multi-family, parent-involved treatment. J Eat Disord.

[19] Thomas JJ, Eddy KT (2019). Cognitive-behavioral therapy for avoidant/restrictive food intake disorder: children, adolescents, & adults.

[20] Thomas JJ, Becker KR, Breithaupt L, Murray HB, Jo JH, Kuhnle MC (2021). Cognitive-behavioral therapy for adults with avoidant/restrictive food intake disorder. J Behav Cogn Ther.

[21] Burton Murray H, Weeks I, Becker KR, Ljótsson B, Madva EN, Eddy KT (2023). Development of a brief cognitive-behavioral treatment for avoidant/restrictive food intake disorder in the context of disorders of gut-brain interaction: Initial feasibility, acceptability, and clinical outcomes. Int J Eat Disord.

[22] Trottier K (2020). Posttraumatic stress disorder predicts non-completion of day hospital treatment for bulimia nervosa and other specified feeding/eating disorder. Eur Eat Disord Rev.

[23] Linehan MM (2015). DBT skills training manual.

[24] Fairburn CG, Cooper Z, O’Connor ME, Fairburn CG (2008). Eating Disorder Examination (Edition 16.0D). Cognitive behavior therapy and eating disorders.

[25] Berg KC, Peterson CB, Frazier P, Crow SJ (2012). Psychometric evaluation of the eating disorder examination and eating disorder examination-questionnaire: A systematic review of the literature. Int J Eat Disord.

[26] Bohn K, Fairburn CG, Fairburn CG (2008). Clinical Impairment Assessment Questionnaire (CIA 3.0). Cognitive behavior therapy and eating disorders.

[27] Maraldo TM, Fewell L, Vander Wal JS (2021). Factor structure and psychometric properties of the Clinical Impairment Assessment 3.0 (CIA) in a clinical eating disorder sample. Eat Behav.

[28] Reas DL, Stedal K, Lindvall Dahlgren C, Rø Ø (2016). Impairment due to eating disorder pathology: identifying the cut-off score on the Clinical Impairment Assessment in a clinical and community sample. Int J Eat Disord.

[29] Weathers FW, Litz BT, Keane TM, Palmieri PA, Marx BP, Schnurr PP. The PTSD Checklist for DSM-5 (PCL-5). 2013. Available from the National Center for PTSD at: www.ptsd.va.govhttps://www.ptsd.va.gov/professional/assessment/adult-sr/ptsd-checklist.asp.

[30] Blevins CA, Weathers FW, Davis MT, Witte TK, Domino JL (2015). The posttraumatic stress disorder checklist for DSM-5 (PCL-5): Development and initial psychometric evaluation. J Trauma Stress.

[31] MacDonald DE, Trottier K, Olmsted MP (2017). Rapid improvements in emotion regulation predict intensive treatment outcome for patients with bulimia nervosa and purging disorder. Int J Eat Disord.

[32] Becker KR, Keshishian AC, Liebman RE, Coniglio KA, Wang SB, Franko DL (2019). Impact of expanded diagnostic criteria for avoidant/restrictive food intake disorder on clinical comparisons with anorexia nervosa. Int J Eat Disord.

[33] Udo T, Grilo CM (2019). Psychiatric and medical correlates of DSM-5 eating disorders in a nationally representative sample of adults in the United States. Int J Eat Disord.

[34] Trottier K, MacDonald DE (2017). Update on psychological trauma, other severe adverse experiences and eating disorders: State of the research and future research directions. Curr Psychiatry Rep.

[35] Burton Murray H, Dreier MJ, Zickgraf HF, Becker KR, Breithaupt L, Eddy KT (2021). Validation of the Nine Item ARFID Screen (NIAS) subscales for distinguishing ARFID presentations and screening for ARFID. Int J Eat Disord.

[36] Reilly EE, Brown TA, Gray EK, Kaye WH, Menzel JE (2019). Exploring the cooccurrence of behavioural phenotypes for avoidant/restrictive food intake disorder in a partial hospitalization sample. Eur Eat Disord Rev.

[37] Bryant-Waugh R, Stern CM, Dreier MJ, Micali N, Cooke LJ, Kuhnle MC (2022). Preliminary validation of the pica, ARFID and rumination interview ARFID questionnaire (PARDI-AR-Q). J Eat Disord.

[38] Becker KR, Mancuso C, Dreier MJ, Asanza E, Breithaupt L, Slattery M (2021). Ghrelin and PYY in low-weight females with avoidant/restrictive food intake disorder compared to anorexia nervosa and healthy controls. Psychoneuroendocrinology.

[39] Menzel JE, Reilly EE, Luo TJ, Kaye WH (2019). Conceptualizing the role of disgust in avoidant/restrictive food intake disorder: Implications for the etiology and treatment of selective eating. Int J Eat Disord.

[40] Roux H, Ali A, Lambert S, Radon L, Huas C, Curt F, Berthoz S, Godart N, the EVHAN Group (2016). Predictive factors of dropout from inpatient treatment for anorexia nervosa. BMC Psychiatry.

[41] Bryant-Waugh R, Micali N, Cooke L, Lawson EA, Eddy KT, Thomas JJ (2019). Development of the Pica, ARFID, and Rumination Disorder Interview, a multi-informant, semi-structured interview of feeding disorders across the lifespan: a pilot study for ages 10–22. Int J Eat Disord.

[42] Zickgraf HF, Ellis JM (2018). Initial validation of the Nine Item Avoidant/Restrictive Food Intake disorder screen (NIAS): a measure of three restrictive eating patterns. Appetite.

[43] Pliner P, Hobden K (1992). Development of a scale to measure the trait of food neophobia in humans. Appetite.

